# Effects of pentoxifylline on the histological and ultra-structural features of vitrified mouse ovarian tissue: An experimental study

**Published:** 2018-06

**Authors:** Elham Aliabadi, Fakhroddin Mesbah, Elias Kargar-Abarghouei, Shahla Zahiri, Shabnam Abdi

**Affiliations:** 1 *Department of Anatomical Sciences, School of Medicine, Shiraz University of Medical Sciences, Shiraz, Iran.*; 2 *Department of Anatomical Sciences and Biology, School of Medicine, Azad University of Medical Sciences, Tehran, Iran.*

**Keywords:** Histology, Mouse, Ovary, Pentoxifylline, Vitrification

## Abstract

**Background:**

Vitrification is a process that can be used to preserve gonads in the healthy and natural status. Oxidative stress is one of the disadvantages of vitrification. Pentoxifylline (PTX) is an antioxidant that can reduce reactive oxidative stress effects.

**Objective:**

We aimed to investigate the effects of PTX on histological and ultra-structural features of vitrified and non-vitrified mouse ovarian tissue.

**Materials and Methods:**

Twenty-five adult female Balb-C mice were randomly and equally divided into control group: the ovaries did not receive any treatment; experimental 1 and 2: the vitrified ovaries were incubated in phosphate buffer solution and bovine serum albumin without and with PTX, respectively, for 30 min; sham 1 and 2: the non-vitrified ovaries were incubated in phosphate buffer solution and bovine serum albumin and were incubated without and with PTX, respectively for 30 min. The right and left ovaries in all of the groups were evaluated using light and transmission electron microscopy, respectively.

**Results:**

The histological and ultra-structural features of vitrified ovaries were seriously damaged. There was non-uniformed germinal epithelium and tunica albuginea, degenerated granulosa cells and stromal cells, puffy basement membrane and irregular thickness of zona pellucida, as well as a pyknotic nucleus and bubbly and segmented ooplasmic in the follicles. Also, ovarian tissues were damaged by the PTX in the non-vitrified ovaries.

**Conclusion:**

Vitrification can damage the histological and ultra-structural features of the ovary in mouse models. PTX as an antioxidant, with concentration of 1.8 mM could not prevent and restore these damages and had no adequate effects on the vitrified ovarian tissues.

## Introduction

Cryopreservation is a natural and artificial phenomenon that can be used to preserve organs, tissues and cells in healthy and natural status against internal and external risk factors. The two major techniques for cryopreservation are conventional slow-freezing and vitrification. The main deficiency of slow-freezing is the formation of ice crystal (1), which leads to serious injury to the tissues and even cell death (2). Although cryoprotectants can be used to eliminate ice formation, toxicity is the greatest disadvantage of these substances. Therefore, the vitrification technique could be used instead to overcome the shortcoming of the slow-freezing technique (3). In vitrification, transmuting tissue liquid directly into the vitreous solidified status prevents the formation of ice crystals (2, 4). The main differences between vitrification and slow-freezing are the rapid cooling and high concentration of subtoxic cryoprotectants (5). Therefore, it can be used for cryopreservation of organs such as the ovary and testis (6). During vitrification, tissue fluids could be replaced by cryoprotectants to prevent the movement of water molecules (2). Cryoprotectants toxicity may be due to osmotic shock, Oxidative stress (OS) and cooling injury (3). Oxidative damage may occur after ovarian vitrification, which could be prevented by anti-oxidant application (3). OS affects cellular components such as proteins, lipids and carbohydrates, leading to cell membrane disintegration and DNA fragmentation (7, 8).

The reproductive biology of females is more challengeable, because of the limited reproductive age, the structural complexity of the ovary, decline in the number of follicles, and high physiological follicular atresia. These properties make the ovaries vulnerable to internal and external risk factors. In order to prevent infertility with ovarian origin in women, cryopreservation has recently been proposed in clinical and experimental protocols. On the other hand, vitrification can overcome the problems of oocyte cryopreservation (2) and increase the ability to obtain many survived oocytes (9), and preserve the ovarian structures and functions (10). 

As mentioned above, the effects of cryoprotectants toxicity may be mainly due to the production of reactive oxidative stress (ROS) which can be resolved by anti-oxidant administration (3). Pentoxifylline (PTX) is a methylxanthine derivative. It also inhibits toxic-free radicals (11). PTX has been used as an antioxidant and can reduce follicular atresia in polycystic ovary syndrome induced rat models (12). 

Some studies have shown that vitrification and cryoprotectants can damage oocyte organelles, zona pellucida (ZP), oolemma and granulosa cells (GCs), which may be protected and reduced by anti-oxidants (13). Therefore, we aimed to investigate the effects of PTX on histological and ultra-structural features of vitrified and non-vitrified mouse ovarian tissue.

## Materials and methods

Adult female (6-8 wk) Balb-C mice (20-30 gr) were randomly selected from the animal lab of Shiraz University of Medical Sciences. 

**Experimental design**

After a vaginal smear test, 25 mice that were in pro-estrus stage were randomly and equally classified into five groups. The mice were sacrificed by cervical dislocation and both ovaries were removed and treated as follows:

Control: the ovaries did not receive any treatment

Experiment 1: the ovaries were vitrified for 2 wk and then warmed and subsequently incubated in phosphate buffer solution (PBS) and bovine serum albumin (BSA) for 30 min

Experiment 2: the ovaries were vitrified for 2 wk and then warmed and subsequently incubated in PBS and BSA supplemented with 1.8 mM PTX (14) for 30 min 

Sham 1: the non-vitrified ovaries were incubated in PBS and BSA for 30min 

Sham 2: the non-vitrified ovaries were incubated in PBS and BSA supplemented with 1.8 mM PTX for 30 min.

**Vitrification of ovarian tissue**

The vitrification solution used in this study contained 40% ethylene glycol (Sigma-Aldrich Company, USA); 30% ficoll 70 (Sigma-Aldrich Company, USA); 0.5 M sucrose (Sigma-Aldrich Company, USA) (15). Intact ovaries were immersed in vitrification solution for 15 min at room temperature. After dehydration, the ovaries were placed into the cryotubes with minimum vitrification medium and directly plunged into liquid nitrogen for 2 wk.

**Warming of ovarian tissue**

The vitrified ovaries were warmed by warming the cryotube in liquid nitrogen vapors for 30 sec, and then immersed in water at 25^o^C until the ice melted. The contents of each cryotube were transferred into 1mL of descending concentrations of sucrose (1, 0.5, 0.25 M) at room temperature for 5 min.

**Ovary incubation**

The warming ovaries were incubated in PBS and BSA without or with 1.8 Mm PTX for 30 min.

**Ovary preparation for light microscopy (LM)**

The right ovary of each mouse was fixed in 10% buffer formalin, dehydrated in a graded ascending series of 30-100% ethanol and embedded in paraffin. 5 μm thick sections were stained with hematoxylin and eosin and observed by LM. The follicles were classified as normal when they contained an intact oocyte and complete layers of GCs. They were considered degenerated when they contained a cytoplasmic micro-vacuolation, pyknotic nucleus, shrunken ooplasm, and/or disrupted GCs.

**Ovary preparation for transmission electron microscopy (TEM)**

The left ovary of each mouse was immersed in 2.5% glutaraldehyde (Sigma-Aldrich Company, USA) for 2-3 hr and then segmented into 1 mm (2). The ovarian segments were primary-fixed in buffered 2.5% glutaraldehyde overnight, washed in sodium cacodylate (Sigma-Aldrich Company, USA) three times/5 min, and then post-fixed in 1% buffered osmium tetroxide (Sigma-Aldrich Company, USA) for 2 hr. Post-fixed segments were washed in sodium cacodylate three times/5 min and high quality distilled water and dehydrated in an ascending series of 30-100% ethanol and 100% acetone. Each segment was embedded in resin (agar 100) and polymerized at 60^o^C overnight. Thick sections (0.5-1 μm) were stained with toluidine blue and examined by LM. Thin sections (60-90 nm), 5 grids of each ovary, were contrasted with uranyl acetate and lead citrate and examined by TEM.

**Ethical consideration**

The animals were housed under controlled conditions (12 hr light/dark at 23±2^o^C), with ad libitum access to food and tap water for 1 wk. The Ethics Committee of the University approved all the animal procedures (2017-355)

## Results

**Histological observations**

**Control group**

The intact germinal epithelium (GE) contained cuboidal and squamous cells with the distinct boundaries. Also, the underlying tunica albuginea (TA) and the spindle shaped stromal cells (SCs) with distinct dark nucleus were seen. The cortical area of the ovary was filled by various types of follicles: primordial, activated, primary, secondary, early antral, few atretic. Most follicles were primordial and contained a defined oocyte with large eccentric nucleus, large nucleolus and normal distribution of ooplasmic organelles. The oocyte was surrounded by intact squamous GCs. The primary follicles were defined and bordered by cuboidal GCs (Figure 2-A). Furthermore, outside the follicular basement membrane (BM) the integrated theca interna and externa were distinguished with long and spindle shaped cells. The secondary follicles with normal oocyte, GCs and BM and theca layers were observed. The atretic follicles showed detached GCs, shrunken oocyte and pyknotic nucleus (Figure 2-A). 

**Experimental groups**

**Vitrified ovaries incubated in PBS and BSA**

Ovaries were covered by GE with cuboidal cells, but degenerated and detached from TA, the TA was not clearly and completely defined (Figure 2-B). Decreased scattered SCs with dark nucleus accompanied with less blood vessels were seen throughout the ovarian stroma (Figure 2-B). The GCs in the primordial follicles contained many vacuoles, the foamy ooplasm had no homogenous distribution of organelles and some oocytes were degenerated (not shown here). There were degenerated GCs and TCs, puffy BM, ZP with irregular thickness, as well as pyknotic nucleus and bubbly and segmented ooplasmin the primary follicles. Furthermore, more atresia of secondary follicles with detached GCs and no clear boundary between theca interna and externa were clearly observed (Figure 2-B). Totally, atretic follicles were more prevalent and CL with many vacuoles were seen in the vitrified ovary.

**Vitrified ovaries incubated in PBS and BSA with PTX**

**Figure 1:**
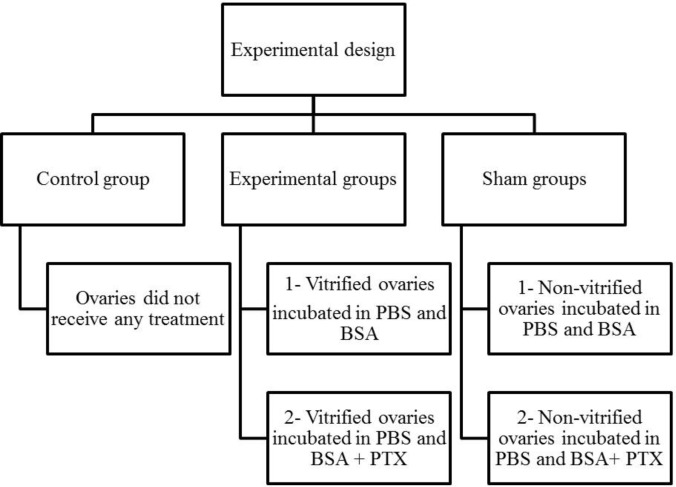
Experimental design, phosphate buffer solution (PBS); bovine serum albumin (BSA); pentoxifylline (PTX).

**Figure 2:**
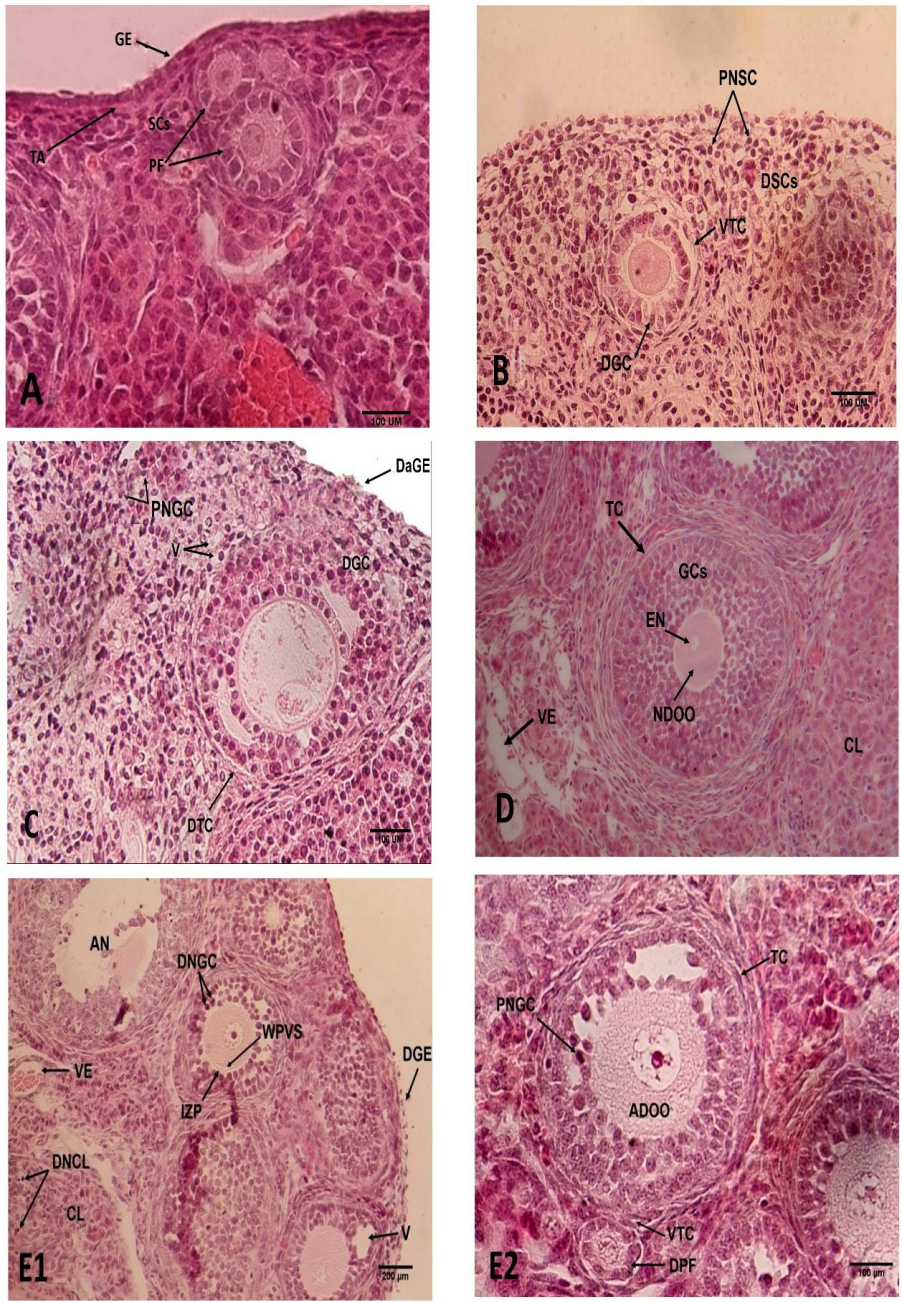
Light micrograph of ovarian tissue (H&E staining).

The GE with cuboidal and squamous cells and their cytoplasmic membrane was unclear and detached from the TA and the TA was damaged. Scattered SCs with dark and round nuclei accompanied with different sized vacuoles were observed (Figure 2-C). More vacuoles were defined in GCs in primordial follicles with degenerated oocyte and GCs, and irregular distribution of organelles in oolema (not shown here). Irregular and proportional thin ZP (Figure 1-C), distorted theca layers and BM, some ruptured and bubbly oocytes with pyknotic nucleus, and also GCs with pyknotic nucleus and destroyed cytoplasm were seen in primary follicles (not shown here). Different shapes of GCs, degeneration of GCs and theca cell layers that were detached from the BM were seen in most of the secondary follicles (Figure 2-C). More atretic follicles, fewer blood vessels and more vacuoles were seen in the stroma than the other groups (Figure 2-C). 

**Sham groups**

**Ovaries Incubated in PBS and BSA**

The intact GE was rested on the TA and contained cuboidal cells. The TA and the spindle shaped SC with distinct nuclei with scarce vacuoles were seen. Normal CL with large luteal cells and vacuoles which represent the pre-estrous phase, many blood vessels and luteinizing thecal cells were observed (Figure 2-D). The ovarian cortex contained primordial follicles, with a normal oocyte and its eccentric nucleus. The oocytes were surrounded by squamous GCs. Primary follicles, with normal GCs without vacuoles, regular ZP thickness and distinct theca layers were seen (not shown here). Theca layers contained elongated and spindle shaped cells and were in close contact with the normal BM. Secondary follicles, with few detached GCs, normal thickness of ZP, BM and theca layers were noticed (Figure 2-D). Few atretic follicles were observed and were more than the control group (not shown here).

**Ovaries incubated in PBS and BSA with PTX**

The ovaries were covered by GE with the cuboidal and squamous cells; some cells were detached from the TA and each other (Figure 2-E1, E2). More vacuoles were seen in the stroma than the control and sham1 groups. Also, CL with more vacuoles were seen, some luteal cells had dark nucleus and more blood vessels were focused in the medulla of ovary. Some primordial follicles were degenerated (Figure 2-E1, E2). In some primary follicles, the thickness of ZP was not regular, theca layers and cells had vacuoles, the early small antrum was defined. In the secondary follicles, the GCs were dark and detached from the BM and the TCs were elongated and spindle-shaped. There were more atretic follicles compared with the ovaries that were incubated in PBS and BSA (sham1) (Figure 2-E1, E2).

**Ultra-structural observations**

**Control group**

**Figure 3:**
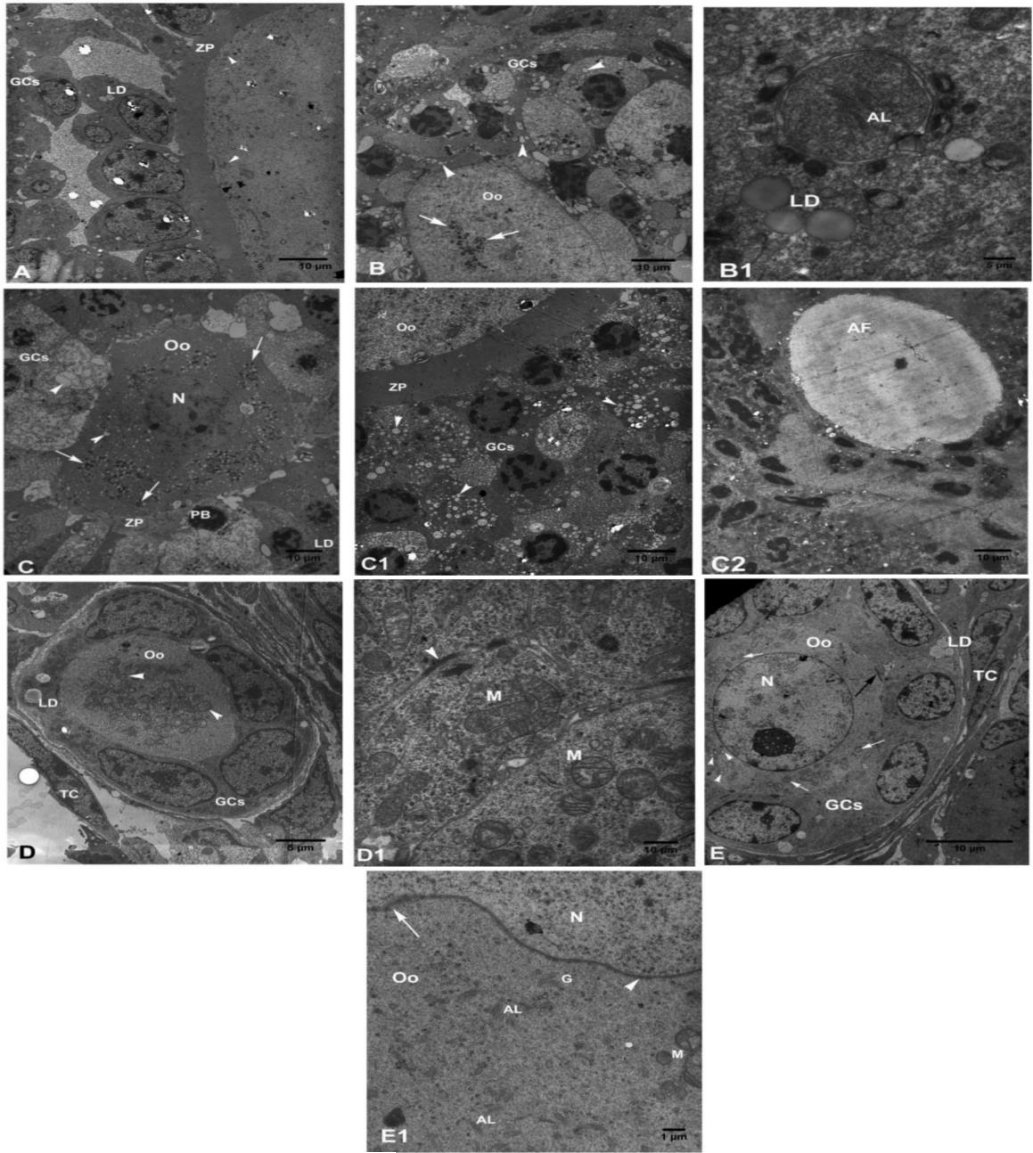
Electron micrographs of the ovarian tissue.

The preantral follicles showed the normal TCs with elongated nuclei in the theca layers, blood vessels were close to theca externa. The theca interna and GCs layer were also separated by the intact BM. The regular GCs with distinct cytoplasmic membrane (Figure 3-A), rounded euchromatin nuclei, nuclear envelope, rough endoplasmic reticulum (RER), dumbly and rounded mitochondria (Mt) with defined cristae (Figure 3-A), and also intercellular junctions between GCs and oocyte and each other’s GCs were observed. The tickness of the ZP was normal, few oocytes had irregular ZP thickness. The oolema, reticular nucleolus and the normal cytoplasmic organelles such as ER and Mt were observed. The Mt with defined cristae were placed peripherally (Figure 3-A).

**Experimental groups**

**Vitrified ovaries incubated in PBS and BSA**

The stromal cells were destroyed and the theca layers and cells were degenerated (Figure 3-B). The pyknotic nuclei, few Mt with non-defined cristae and a large number of vacuoles in various sizes were also seen in the TCs. The BM in some areas had disappeared and was found to be irregular. The cytoplasmic GCs membrane was not clearly defined and the boundary between the cells was not determined. In most GCs, the cytoplasmic organelles were not normally distributed and some organelles were completely destroyed (Figure 3-B). There were low RER, smooth endoplasmic reticulum (SER), small size Mt with non-obvious cristae and large numbers of vacuoles in the cytoplasm of these cells were observed (Figure 2-B). Cell junctions between GCs were not observed. The nuclei of GCs were pyknotic and their nuclear envelopes were not visible in all the cells. In some follicles, the ZP thickness was reduced and contained microvilli (MV) and cumulus cells process endings (CCPE). The oolema was not clearly visible in some oocytes. Ooplasmic organelles were scattered, but a few were clustered. The numbers of rounded Mt with non-defined cristae were in close contact with vacuoles, as well as few RER and lipid droplets (LD) and phagosome-like structures containing annulate lamellae (AL) were seen (Figure 3-B, B1).

**Vitrified ovaries incubated in PBS, BSA with PTX**

The theca layers were not clearly seen and the TCs contained vacuoles. The blood vessels were not found near the theca externa and BM was not observed in all the follicles. Two-layers of cytoplasmic membrane in the GCs were not clearly observed and no identified boundary was seen between the cell membrane. Also, some GCs were destroyed. Some cytoplasmic organelles had diminished, but few RER, LD, and Mt were observed (Figure 3-C). The Mt had non-obvious cristae and some cristae were decomposed and swollen. Intercellular junctions between the GCs were not observed and some GCs had pyknotic nuclei (Figure 3-C, C1). The irregular ZP with different thickness in various oocytes was observed and the ZP did not contain MV and CCEP. The oolemma was not clearly defined and PVS was not seen. Most ooplasmic organelles were dispersed with irregular distribution. The RER, Mt with non-defined cristae and a large number of vacuoles were seen in the ooplasm (Figure 3-C, C1, C2).

**Sham groups**

**Ovaries incubated in PBS and BSA**

Few SCs were destroyed, but distinct theca layers and normal BM were noticed, theca cell organelles including Mt were normally distributed throughout the cytoplasm (Figure 3-D). Some blood vessels were closed to the theca externa. The GCs had normal features of a cell membrane and also contained RER, SER, Mt, and LD, but few pyknotic nuclei (Figure 3-D, D1). Some Mt with swelling cristae and vacuoles were also seen (Figure 3-D1). The ZP was observed in all the follicles except primordial follicles and contained many CCEP and MV and ZP was thick in some oocytes. The normal distribution of ooplasmic organelles such as the clusters of rounded Mt with defined cristae, few RER and AL were noticed. Reticular nucleolus and bilayer of nuclear envelope of oocyte were seen (Figure 3-D, D1).

**Ovaries incubated in PBS and BSA with PTX**

There were theca interna and externa and TCs with elongated and rounded nuclei. Many Mt with distinct cristae were seen in the cytoplasm of TCs (Figure 3-E, E1). Other organelles were evenly distributed throughout the cytoplasm, and a cluster of RER and vesicular SER were defined. Some TCs did not clearly show cytoplasmic organelles and contained some vacuoles. The cytoplasmic organelles of GCs include rounded, dumbly and elongated Mt with distinct cristae, SER, RER, Golgi complex, and LD were normally distributed. Some vacuoles were seen in GCs. In some GCs, the nucleus was deformed and lobulated. The oocytes with normal oolema and normal ZP thickness containing MV and CCPE were observed. There was no PVS in follicles with irregular ZP thickness. Aggregated organelles in the ooplasm were seen like the clusters of Mt, RER and SER, Golgi complex close to the nuclear envelope and AL. The two-layer membrane of the nuclear envelope with its pores and reticular nucleolus was observed in the oocyte (Figure 3-E, E1).

A), Control group, showing the normal germinal epithelium (GE), tunica albuginea (TA), primary follicle (PF), stromal cells (SCs); B), Experimental 1, displaying the degenerated stromal cells (DSCs), vacuole in theca cells (VTC), degenerated granulosa cells (DGCs), pyknotic nuclei stroma cell (PNSC); C), Experimental 2, pyknotic nuclei granulosa cell (PNGC), vacuole(V), damaged germinal epithelium (DaGE), DGCs, degenerated theca cells (DTC); D) Sham1, ovarian tissue showing, corpus luteum (CL), theca cells (TC), vessel(VE), eccentric nuclei (EN), normal distribution oocyte organelle (NDOO), GCs; E1 and E2) Sham2, displaying DGE, antrum (AN), VE, dark nucleolus carpus luteum (DNCL), IZP, dark nucleolus granulosa cell (DNGC), wide perivittiline space (WPVS), PNGC, ADOO, V in theca cell, degenerated primary follicle (DPF), theca cell V between granulosa cells (V) and atretic oocyte (AO), degenerated nucleus (DN).

A) Control group, showing granulosa cell (GCs) with lipid droplets (LD), zona pellucid (ZP) contains GCs processes and oocyte microvilli, mitochondria (arrowhead);

B and B1) Experimental1, oocyte (Oo) with mitochondria (arrow) and non-obvious ZP that surrounded by degenerated and vacuolated (arrowhead) GCs. annulate lamellae (AL);

C, C1 and C2) Experimental2, irregular shaped Oo which surrounded by degenerated GCs and pyknotic body (PB), contains dark mitochondria (arrow), numerous vacuole (arrowhead) and nucleus (N) with a patch of ZP, an Oo with normal ZP which surrounded by degenerated and vacuolated (arrowhead) GCs (in C1) and a completely atretic follicle (AF) with collapsed ZP (in C2);

D, D1), Sham1, a normal activated follicle with normal theca cell (TC) and Oo which contains obvious mitochondria (arrowhead in D), GCs with rounded, hocked and dumbbell shaped obvious mitochondria (M) and junctions between granulose cells (arrowhead in D1);

E, E1) Sham2, a normal activated follicle with TC, GCs contains LD, normal Oo with nucleus (N) and reticular nucleolus, mitochondria (arrow), vacuoles (arrowhead) and a visible junction between oocyte and granulose cell (dark arrow), part of Oo that showing N with clear ((arrowhead) and non-clear (arrow) nuclear envelope (in E1) and, Golgi apparatus (G), AL and M.

## Discussion

To protect the oocyte and/or ovary against internal and external harmful factors, cryopreservation is an appropriate technique, which helps to preserve the fertility capabilities of the oocyte and ovary (16). There are some controversial decisions about cryopreservation either for the oocyte or ovary. Preservation of immature oocyte is more appreciated than the MII oocyte (17), because cryopreservation-derived injury to the meiotic spindle in the matured oocytes leads to aneuploidy, however, the immature oocyte has no meiotic spindle (18). Therefore, it seems that cryopreservation of ovarian tissue may be more favorable, since it maintains the normal features of the oocyte and distribution of ooplasmic organelles and oolema proteins. 

Previous study has shown that vitrification is a more appropriate technique than slow-freezing for oocyte preservation (19). But, we found that many ooplasmic organelles and oolema as well as cell membrane and cytoplasm of GCs and TCs were destroyed in the vitrified ovary at both LM and TEM levels. TEM observation is a gold standard method for evaluating ultra-structures and the quality of vitrified oocyte. Alteration in ooplasmic organelles, cell membrane and ZP in ovarian tissue represents the physiological and pathological changes in the ovaries. Early appearance and exocytosis of cortical granules (CGs) may result in hard ZP leading to reduced fertilization (20), however, it may be decreased if low concentrations of cryoprotectants are applied (3). The ZP as a glycoprotein structure prevents polyspermy and protects blastocysts, which may be damaged during cryopreservation, thus, changes in normal texture of ZP may lead to decrease fertility rate (21).

The intact GCs can prevent OS caused by ice crystal formation of oocyte during cryopreservation. We found damaged and dispersed GCs, and also irregular ZP thickness with abnormal texture and early appearing CGs, which were not only observed in vitrified ovaries incubated in PBS and BSA, but also in the ovaries treated with PTX with or without vitrification. The previous findings in cryopreserved human oocyte are consistent with our results (13). Also, it is reported that thermal shock may lead to parthenogenesis activation during cryopreservation (18). Movement of water molecules between inside and outside of the primordial oocytes may be reduced because of the small size of the oocyte, thus the damages of ice formation is decreased in these oocytes, as we have seen in primary and secondary follicles (18). The vitrification itself can also prevent ice crystal formation, because it is a rapid technique (19, 22). 

The cryopreservation of ovarian tissues is preferred than the mature oocyte; however, some technical stages of cryopreservation may be also harmful such as ice crystal formation, as shown in the results of our study (18). Moreover, Tao and Del Valle reported that oocyte cryopreservation is better than embryo cryopreservation for fertility preservation (23). Even though ovarian cryopreservation is used for transplantation, certainly one of the main problems is its revascularization. However, ovarian cryopreservation either by slow freezing or vitrification resolves some issues caused by oocyte cryopreservation (2). In contrast with previous finding,our results showed obvious damages in oocyte, GCs, ZP, oolema and TCs in vitrified ovary both under LM and TEM, and these changes are so much that PTX cannot inhibit them (21, 24). We found more vacuoles in vitrified ovaries that were incubated in both supplemented media without and with PTX and rarely in non-vitrified ovaries which were incubated in supplemented media with PTX. These results can cause reduced fertility efficiency in ovary and oocyte (19).

Although, there are contradictory conclusions regarding the Mt status in vitrified oocyte in human and other mammals, different shapes of damaged Mt with no obvious cristae have been observed in our study, despite the fact that we used ethylene glycol in in this experiment (25-27). The abnormal distribution of organelles in our study in experimental ovaries in the presence or absence of PTX in culture media may lead to infertility. Reduction in the density of MV and CCPE in the oocyte both in the absence or presence of PTX may result in fertilization failure and loss of integration of the sperm-oocyte (28).

Although it is clearly known that OS has destructive effects on carbohydrates, lipids and proteins of cells membrane (29), and PTX is an anti-oxidant which reduces OS and ROS derived from Mt (11) the tissues can be damaged as a result of osmotic shock and OS during vitrification (5). We found that the more obvious destruction of ovarian and follicular structures includes GE, TA, SC, TC, GCs and oocytes at histological and ultra-structural levels in PTX-treated ovaries both in vitrified and non-vitrified groups. These results demonstrate that PTX not only prevents and/or restores tissue damage, but also injures the ovarian tissue in the non-vitrified group. This could be attributed to the inadequate duration of incubation with PTX, insufficient concentration of PTX, high toxicity of cryoprotectants, as well as the side effects of PTX (30). 

Moreover, if vitrification is carried out in one stage, it may cause more damage to the tissues compared with multi-stage vitrification, as done in our study (31). Using the multi-stage vitrification method gives the opportunity to the tissue to regulate permeability of cell membrane, in order to maintain cell junctions. Another reason for ineffectiveness of the PTX to prevent damage to PTX-treated ovary in this study, is that PTX was used locally, but not systemically. The oral administration of PTX reduced OS in letrozole-induced PCO in rat model, and maintained normal histological texture of follicles (32, 12). Moreover, PTX causes mitochondrial and nuclear destruction, because the time of DNA repair can be affected by PTX, therefore it can induce the apoptosis in cells, which leads to damage the mt and nucleus (33). Thus it is not unexpected to observe more destruction in PTX-treated ovary especially both in vitrified and non-vitrified ovary in this study. On the other hand, it might be useful if PTX is used concurrently with vitrification as a cryoprotectant.

## Conclusion

Vitrification can damage the histological and ultra-structural features of the ovary in mouse models. PTX, as an antioxidant, could not prevent and restore these damages and had no adequate effects on the vitrified ovarian tissues. Observation of the ultra-structural features of the ovary after cryopreservation gives us the opportunity to know more detailed information about ovarian morphological abnormality, dysfunctions and cellular responsibility of fertility deficiency. To find more details on the impact of vitrification and PTX on ovarian tissue, simultaneous administration of PTX with cryoprotectants; use different concentrations of PTX; application of histochemical methods alongside TEM technique are suggested.

## Note

***This article extracted from of MSc. thesis. (Elias Kargar Abarghouei)***
